# Plane wave versus focused transmissions for contrast enhanced ultrasound imaging: the role of parameter settings and the effects of flow rate on contrast measurements

**DOI:** 10.1088/1361-6560/ab13f2

**Published:** 2019-04-23

**Authors:** Elahe Moghimirad, Jeffrey Bamber, Emma Harris

**Affiliations:** pmbab13f23 Joint Department of Physics and CRUK Cancer Imaging Centre, The Institute of Cancer Research and Royal Marsden NHS Foundation Trust, Sutton, London, United Kingdom; 1 The Institute of Cancer Research, 15 Cotswold Road, Sutton, SM2 5NG, United Kingdom; 2 Author to whom any correspondence should be addressed.; Emma.Harris@icr.ac.uk

**Keywords:** contrast enhanced ultrasound imaging, contrast agents, microbubbles, focused imaging, plane wave imaging, grating lobes, flow speed

## Abstract

Contrast enhanced ultrasound (CEUS) and dynamic contrast enhanced ultrasound (DCE-US) can be used to provide information about the vasculature aiding diagnosis and monitoring of a number of pathologies including cancer. In the development of a CEUS imaging system, there are many choices to be made, such as whether to use plane wave (PW) or focused imaging (FI), and the values for parameters such as transmit frequency, F-number, mechanical index, and number of compounding angles (for PW imaging). CEUS image contrast may also be dependent on subject characteristics, e.g. flow speed and vessel orientation. We evaluated the effect of such choices on vessel contrast for PW and FI *in vitro*, using 2D ultrasound imaging. CEUS images were obtained using a Vantage^TM^ (Verasonics Inc.) and a pulse-inversion (PI) algorithm on a flow phantom. Contrast (C) and contrast reduction (CR) were calculated, where C was the initial ratio of signal in vessel to signal in background and CR was its reduction after 200 frames (acquired in 20 s). Two transducer orientations were used: parallel and perpendicular to the vessel direction. Similar C and CR was achievable for PW and FI by choosing optimal parameter values. PW imaging suffered from high frequency grating lobe artefacts, which may lead to degraded image quality and misinterpretation of data. Flow rate influenced the contrast based on: (1) false contrast increase due to the bubble motion between the PI positive and negative pulses (for both PW and FI), and (2) contrast reduction due to the incoherency caused by bubble motion between the compounding angles (for PW only). The effects were less pronounced for perpendicular transducer orientation compared to a parallel one. Although both effects are undesirable, it may be more straight forward to account for artefacts in FI as it only suffers from the former effect. In conclusion, if higher frame rate imaging is not required (a benefit of PW), FI appears to be a better choice of imaging mode for CEUS, providing greater image quality over PW for similar rates of contrast reduction.

## Introduction

Measures of vascularity, for example, blood volume or perfusion are valuable diagnostic markers in a wide range of pathologies and are potential indicators for tumour response to therapies (Frohlich *et al* 2015). For example, chemoradiation can significantly affect the blood perfusion and volume soon after treatment and can be used to differentiate between responders and non-responders (Nishimura *et al* 2016, Tawada *et al* 2009). Also, tumour vasculature is the target for novel antiangiogenic and antivascular therapeutic strategies. An accurate and reproducible technique to quantify changes in tumour vascularity quantitatively at early time points may allow us to improve the way we treat patients (Dietrich *et al* 2012, Jayson *et al* 2016).

Contrast-enhanced ultrasound (CEUS) imaging using microbubble contrast agents, studied since the 1970s, is recognised as a useful tool to study the vasculature (Liu *et al* 2005). Dynamic contrast enhanced ultrasound (DCE-US) extends CEUS by providing the change in contrast signal with time at each image location as microbubbles flow into, and wash out of, the imaged region, known as a time-intensity curve (TIC) (Fleischer *et al* 2004). Both CEUS and DCE-US can measure characteristics related to the function of the vasculature.

We propose to use CEUS and DCE-US to monitor tumour response to chemoradiotherapy and to evaluate tumour perfusion based on vascular density and morphology, and characteristics of the corresponding TICs. Accurate, precise and reproducible quantification of CEUS and DCE-US characteristics is affected by imaging mode, system parameters and subject variables (Tang *et al* 2011). Although, several groups have studied CEUS imaging variability with respect to one or two system parameters including pulse length, frequency, mechanical index (peak negative pressure / square root of transmit frequency) and imaging modes (Vaka 2011, Couture *et al* 2012), others have mostly focused on scanner parameters such as dynamic range, gain, compression, TIC fitting or region of interest (ROI) selection (Lucidarme *et al* 2003, Ignee *et al* 2010, Tang *et al* 2011, Gauthier *et al* 2011).

Plane wave (PW) imaging and conventional focused imaging (FI) are the two well established modes for ultrasound imaging. PW imaging is known for its rapid data acquisition, potentially making it an attractive choice if we wished to extend the system to 3D imaging of temporally-varying contrast agent concentration, i.e. for 3D DCE-US measurement of the change in contrast signal within a given volume of interest, faster imaging will allow greater temporal sampling for generation of TICs. The use of 3D imaging is reported to increase reproducibility of the measured contrast ultrasound characteristics between imaging sessions compared to 2D, thereby improving sensitivity to therapy-induced changes in the vasculature (Liu *et al* 2005, Feingold *et al* 2010, Hoyt *et al* 2012a, 2012b, Mahoney *et al* 2014, Wang *et al* 2015).

Contrast and spatial resolution of B-mode (non-contrast) ultrasound images generated using PWs have been shown to be equivalent to those obtained with monofocused and four-focus FI when using coherent compounding of multiple PW images acquired at 12 or 43 angles, respectively, whilst providing a factor of ten increase in frame rate (Montaldo *et al* 2009). Further improvements in PW contrast have been reported after application of coded coherent plane wave compounding, referred to as multiplane wave imaging (Tiran *et al* 2015). Contrast increases between 2–6 dB compared to non-encoded plane wave imaging were observed and found to depend on the depth of the contrast target and the number of compounding angles used. This same approach was adopted by Gong *et al* (2018) to improve contrast for PW imaging of ultrasound contrast agent using amplitude modulation. Contrast was increased by approximately 5 dB using coded coherently compounded PW imaging compounded to its non-encoded counterpart, comparisons with FI were not made.

Multi-pulse sequences, commonly used to suppress tissue non-linear signals from tissues and include pulse inversion and amplitude modulation, suffer from degradation of contrast caused by tissue and bubble motion due to blood flow affect both PW and FI (Lefort *et al* 2012, Denarie *et al* 2013, Lin *et al* 2013, Nie *et al* 2014, Stanziola *et al* 2018). Artefacts, caused by bubble motion between modified pulses, have been characterised for FI in one study (Lin *et al* 2013). For PW imaging additional image degradation is caused by tissue and bubble motion between acquisitions acquired at different angles and increases with increasing number of angles or time between acquisitions at each angle. This was demonstrated *in vitro* by Viti *et al* (2016) who detected a reduction in contrast when the speed of out of plane (elevational) flow in a vessel phantom was increased from 20 mm s^−1^ to 55 mm s^−1^ for 31 and 63 angles, but not for 15 angles or less. In the field of echocardiography, high flow speeds and cardiac tissue motion, can significantly reduce contrast. Stanziola *et al* (2018) used simulations to show that increasing the number of compounding angles (up to 11) and axial bubble motion (up to 500 mm s^−1^) reduced contrast by up to 28.7 dB. Motion (tissue and bubble) compensation techniques using image registration and cross-correlation can partially correct for motion for *in vivo* imaging acquired using ultrafast diverging waves (DW) combined with pulse inversion and amplitude modulation (Nie *et al* 2014) pulse-sequences.

Whilst there have been studies of the two imaging modes, there is a scarcity of studies that make direct comparison between them. The competing benefits and drawbacks of FI or PW imaging for CEUS imaging are not well understood, nor are the effects of choices for system parameters for each imaging mode. To the best of our knowledge only two groups have reported on the direct comparison of FI and PW CEUS *in vitro*, both used amplitude modulation of the ultrasound pulse sequence to detect the microbubbles (Couture *et al* 2012, Viti *et al* 2016). A third study compared FI and coherently compounded diverging wave images acquired using 11 angles of a sheep’s heart *in vivo* and found a 2 dB improvement in contrast using PW (Toulemonde *et al* 2016). Unanswered questions remain on how to choose parameters such as F-number (F#  =  focal distance / aperture width), MI, transmit frequency, number of compounding angles and, most importantly, how imaging mode (PW or FI) affects the CEUS image and the variability of its quantitative characteristics.

To extend the current knowledge in the field and as a first step towards developing a CEUS imaging system, the aim of this work was to evaluate CEUS image contrast variation using a different pulse sequence, wider range of parameter settings and flow rates, and finally two different vessel orientations. The evaluations were performed using a pulse inversion technique and 2D ultrasound imaging *in vitro*, with respect to (a) imaging modes (PW and FI) and their corresponding parameters, and (b) subject characteristics such as flow speed and vessel orientation.

## Materials and methods

**Figure 1.**
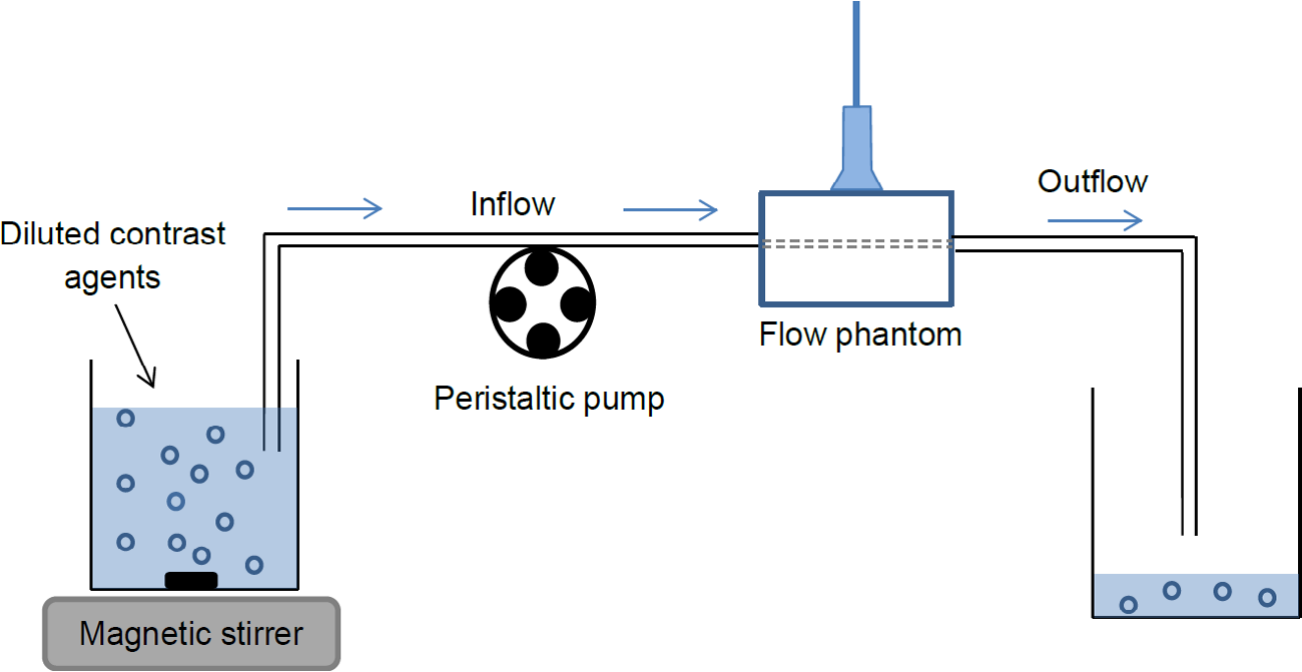
Phantom setup composed of a flow phantom connected to a peristaltic pump. (Contrast agent pumped from the source container was removed from the system after passing through the flow phantom.)

The study was performed using the tissue mimicking flow phantom model 524 (ATS Laboratories Inc, Norfolk, VA, USA) with 0.5 dB/cm/MHz attenuation coefficient and contains four flow channels of 2, 4, 6, and 8 mm diameter located 15.0 mm below the scan surface. The 4 mm diameter vessel was used connecting to a H.R. flow inducer MHRE 200–250 v peristaltic pump (Watson Marlow limited, Falmouth, Cornwall, UK) as shown in figure 1. CEUS images were obtained using the Vantage^TM^ system (Verasonics Inc., Kirkland WA, USA) coupled with an ATL L7-4 transducer (Philips Co., Amsterdam, Netherlands) and reconstructed using a two-pulse pulse inversion algorithm with 0.15 ms pulse intervals. 0.15 ms is chosen to cover the whole phantom depth (imaging depth  =  11.5 cm) without interference (this is also a realistic depth for clinical imaging). The intervals between line and angle transmissions for FI and PW imaging were kept constant at 0.3 ms (=2 $\times$ 0.15 ms) and so the acquisition time for one frame is 38.4 ms and 2.1 ms for 128 lines FI and seven angles PW, respectively. The time to acquire next frame was adjusted to achieve 10 Hz frame rate for both methods and for all experiments. A constant frame rate was chosen for both FI and PW imaging to make sure that the environmental effects on the microbbuble behaviour such as natural degrading, floating or settling are similar during the acquisition of multiple frames.

One hundred and twenty eight beam lines were transmitted for FI, focused at 20 mm depth, approximately equal to the transducer elevational focus. PW imaging was also performed for various number of compounding angles (3, 7, 11, 23 and 35 angles), tilted between  −10 and  +10 degree. The contrast agent (Sonozoid^TM^; GE Healthcare, Oslo, Norway), diluted to a 1:2500 concentration, was pumped into the flow phantom for each experiment and constant concentration was maintained. Sonozoid was chosen because of its relatively good stability, which assisted its use in phantom studies and in experiments where bubbles are assumed to be less stable than *in vivo*.

**Figure 2.**
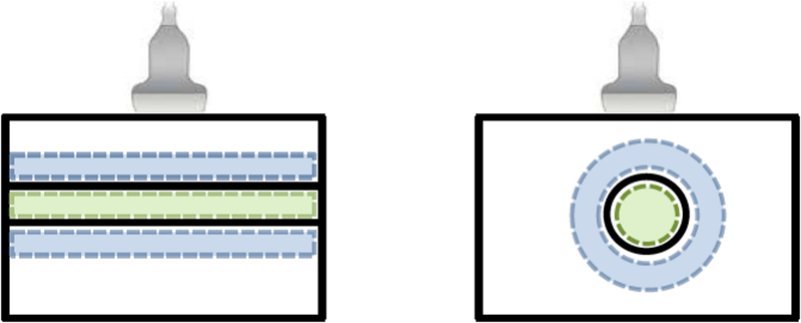
Schematics of the two transducer positions (left) parallel, and (right) perpendicular (cross-sectional). Vessel and background regions are highlighted in green and blue, correspondingly. Having the vessel diameter of 4 mm and considering a small margin, most restricted dimension of the vessel ROIs and accordingly other ROIs was selected as  ∼3.4 mm.

For both imaging modes, FI and PW, a microbubble-specific signal was generated using a standard two-pulse pulse-inversion sequence to suppress the linear tissue signal (Simpson *et al* 1999). Contrast (C) and contrast reduction (CR) were used as the evaluation criteria, where C was the initial ratio of mean signal in the vessel to mean signal in the background measured on the first frame and CR was the reduction in contrast after 200 frames (a measure of bubble disruption). The experiments were performed for two different transducer orientations, parallel and perpendicular with respect to the vessel. The corresponding vessel and background ROIs are shown schematically in figure 2. Two sets of experiments were defined as: (a) ‘parameter settings’ to evaluate the effect of different parameters for the two imaging modes and (b) ‘flow effects’ to evaluate the influence of flow on FI and PW contrast imaging.

### Parameter settings

To evaluate the effect of the parameters on contrast, several values of transmit frequency, MI, F#, and number of coherent compounding angles (for PW imaging only) were studied for PW and FI. For each experiment, the pump was turned off after pumping a fresh set of bubbles into the vessel and then 200 frames (recorded in 20 s) were acquired for FI and PW. To evaluate the parameters independently, one parameter was change at a time where other parameters were set to: frequency  =  4 MHz, F#  =  4, MI  =  0.15, seven angles and zero flow. The transducer was initially oriented in the parallel direction and the parameters were changed using the values in table 1. The minimum MI and frequency were dictated by system and transducer limitations. All measurements were repeated a minimum of three times. The experiment was then performed with the transducer orientated perpendicular to the vessel direction. In this case, artefacts discovered for PW imaging required different background ROIs to measure their effect on the contrast (see figure 5(b)). To further study the artefacts, low-pass filters with 5 MHz, 7 MHz and 9 MHz cut-off frequencies were applied to the receive data in the perpendicular orientation (filtering was applied to the channel data acquired for each line and each steering angle, prior to image beamformation) with various transmit frequencies and the corresponding image contrasts were evaluated to understand the frequency dependency of the artefact.

**Table 1.** Parameter settings.

Parameter	Values
Transmit frequency (MHz)	3, 4, 5
Mechanical index	0.11, 0.15, 0.25
F#	2, 3, 4
No. of compounding angles	3, 7, 11, 23, 35

### Flow effects

To study how the flow rate affects contrast, the results of the parameter setting experiments were used to set F#, MI, transmit frequency and number of PW angles to achieve the same C and CR for both FI and PW imaging. The parameters were: frequency  =  4 MHz, F#  =  4, MI  =  0.15 and seven angles. The flow speed was varied by adjusting the flow rate of the peristaltic pump. The following flow speeds were used (in mm s^−1^): 0, 54, 80, 103, 133, 159, 212, 265, 379 and 504, and C was evaluated for FI and PW imaging. These speeds included the range of flow speeds found in the human body, ∼0.3 mm s^−1^ in capillaries to  ∼400 mm s^−1^ in aorta (Marieb and Hoehn 2013). Two pulse intervals of 0.15 ms and 0.3 ms were used for pulse inversion and the number of angles for PW imaging was also changed to 3, 7, 11 and 23 angles, as the variation in contrast with flow rate was hypothesised to be highly dependent on these two parameters for PW and FI. All other parameters were kept constant. To compare the flow effect in parallel and perpendicular orientation, the measurements were repeated with the transducer perpendicular to the direction of flow for FI and PW imaging with seven angles. All measurements were repeated a minimum of three times.

## Results

### Parameter settings

**Figure 3.**
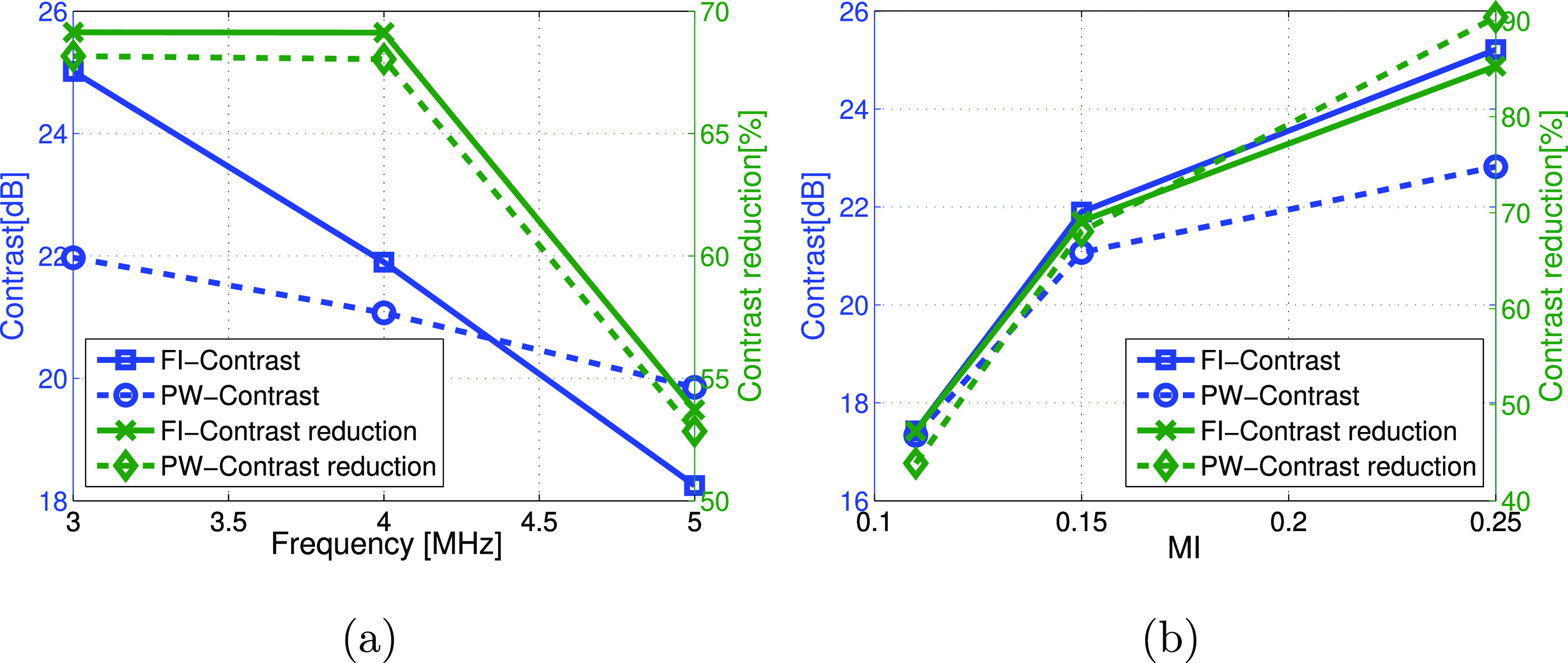
Vessel-to-background contrast and contrast reduction are compared for focused versus PW (seven angles) imaging as a function of (a) frequency (MI  =  0.15), and (b) MI (4 MHz transmit frequency). Maximum deviation between repeat measures was 0.5 dB for contrast and 1.3% for contrast reduction.

C and CR were evaluated for various parameters at zero flow rate and the two imaging modes. They are plotted as a function of frequency in figure 3(a). PW imaging had the greatest contrast at 5 MHz with similar CR compared to FI where 3 MHz gave a higher contrast. The contrasts were comparable at 4 MHz. Figure 3(b) shows C and CR as a function of MI, illustrating that CR increased with MI for both imaging modes but the rate of the CR change was greater for PW imaging compared to FI. Also, FI had 2 dB greater contrast over PW at an MI of 0.25 while they were comparable at MIs of 0.11 and 0.15.

Evaluation of FI contrast for different F#’s (not shown here) showed 5 and 6 dB greater contrast for F3 and F4, respectively, compared to F2, with approximately 20% more CR. PW imaging was also evaluated for various numbers of compounded angles (not shown here), showing that a greater contrast was achieved with more compounding angles at the cost of higher contrast reduction. Using 35 angles resulted in 6 dB greater contrast but 40% more bubble disruption compared to seven angles.

**Figure 4.**
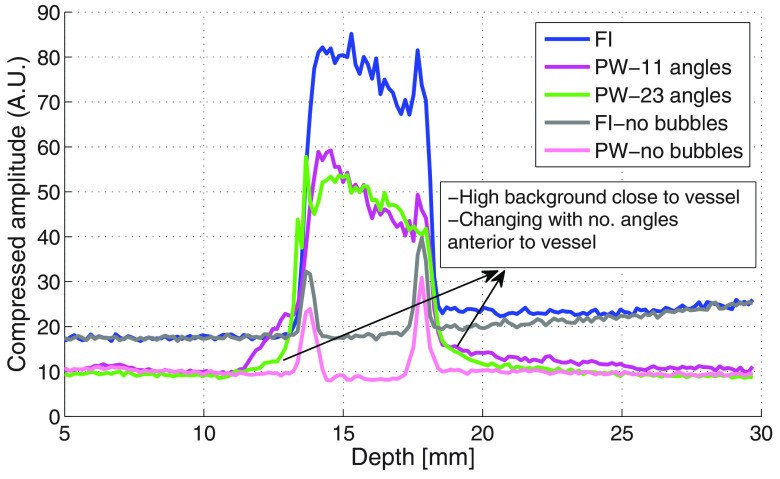
Vessel axial profile for FI and PW imaging with 11 and 23 angles and transducer in parallel direction (frequency  =  4 MHz, MI  =  0.15). The profiles in the absence of contrast are included for comparison. Square root compression was used to illustrate the total dynamic range.

PW and FI achieved similar results of C $\simeq$ 21 dB and CR $\simeq$ 68% at frequency  =  4 MHz, MI  =  0.15, F#  =  4 and seven angles. However, a disadvantage for PW imaging was the effect of side lobes which was observed at the edge of the vessels when the transducer was in the parallel direction. They increased the background signal close to the vessel, reducing the sharpness of the vessel edge. Figure 4 gives axial profiles through the vessel and illustrates how the vessel edge is less sharp for PW imaging compared to FI. The effect of side lobes on the image anterior to the vessel reduced with increasing number of angles for PW. It was, however, still noticeable compared to the vessel profile when there was no contrast agent present.

**Figure 5.**
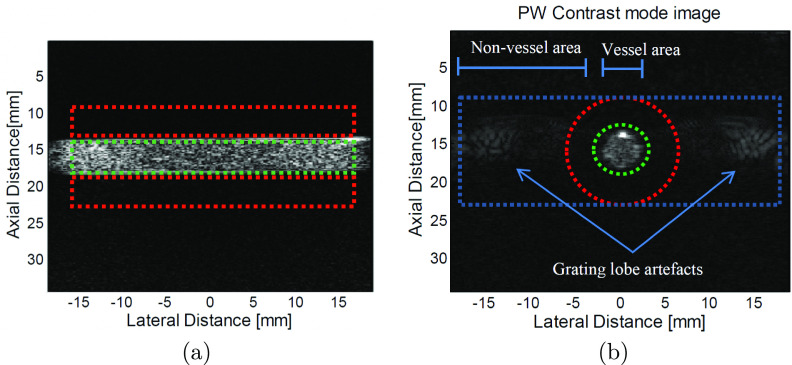
(a) A parallel PW (seven angles) pulse-inversion image showing contrast in the vessel. Vessel and background ROIs are shown in green and red, respectively. (b) A perpendicular PW (seven angles) pulse-inversion image showing contrast in the vessel, grating lobe artefacts and two alternative selections for the background ROI used to investigate the artefacts (ROI1: green and red as inner and outer boundary, respectively, ROI2: green and blue as inner and outer boundary, respectively); the vessel ROI (not shown) is inside the centre contrast region with 3.4 mm diameter. Mean axial profiles were generated by averaging axial lines within the vessel area (3.4 mm width) and the non-vessel area (13 mm width). Note that artefacts were not clearly visualised in the parallel images as they lay inside the vessel.

**Figure 6.**
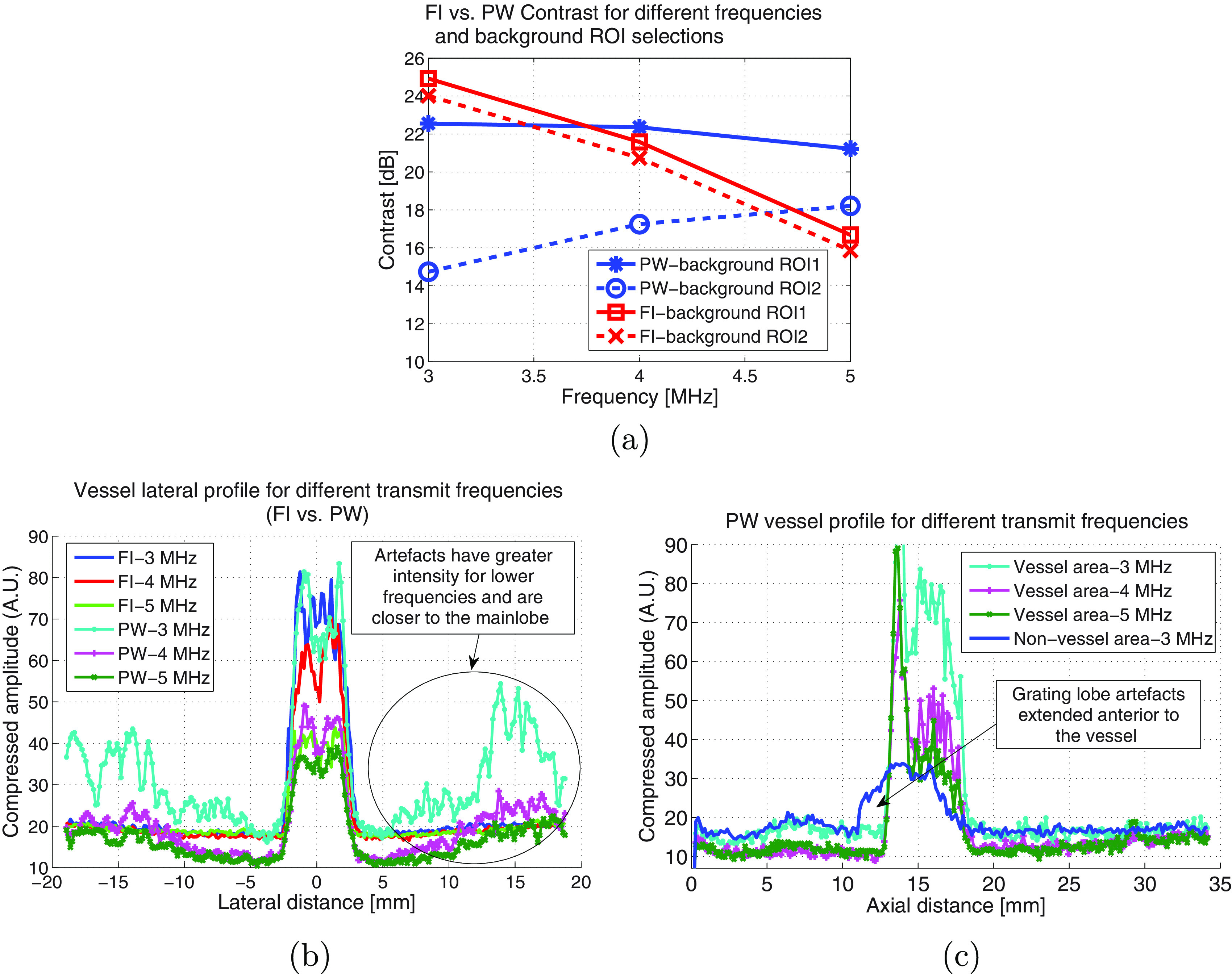
(a) The effect of varying frequency and background ROI on contrast for PW and FI, (b) vessel lateral profiles for PW and FI for different transmit frequencies, and (c) PW imaging axial profile for two areas shown as vessel area and non-vessel area in (a). Square root compression was used for image illustration in (a) and its corresponding lateral and axial profiles in (c) and (d).

In the perpendicular orientation, a pattern of increased intensity, very similar to grating lobe artefacts, was present for PW imaging at a lateral distance about $\pm$15 mm from the vessel centre (figure 5(b)). These artefacts were not evident in the parallel direction (figure 5(a)). To evaluate the effect of these artefacts, which we refer to as grating lobe artefacts, on vessel contrast, two different background ROIs were selected as shown in figure 5(b), and the contrasts are shown in figure 6(a) for different frequencies. These grating lobes artefacts were found to reduce contrast for PW imaging by 7 dB at 3 MHz, and 3 dB at 5 MHz. The artefacts were not seen for FI. The vessel lateral profile is shown in figure 6(b) and illustrates the grating lobes artefacts with respect to the vessel signal for different frequencies and compares it to the FI which has no grating lobe artefacts. The axial image profiles for PW are also shown for different frequencies in figure 6(c) averaged over the vessel area and the non-vessel area. It illustrates greater background noise at 3 MHz compared to 4 MHz and 5 MHz over the whole image.

We hypothesised that these grating lobe artefacts were caused by the high frequency content of the contrast signal generated more at transmit frequencies closer to the microbubble resonance frequency (around 3 MHz). This hypothesis was tested by applying different low-pass filters. As the cut-off frequency reduced, the artefacts were observed to shift away from the vessel centre and decrease in intensity (not shown here). This observation showed the effect of high frequency contrast signal on grating lobe artefacts and supports the hypothesis. The filters, however, also removed the actual high frequency contrast signal and as a result, no contrast gain was achieved using these filters.

### Flow effects

**Figure 7.**
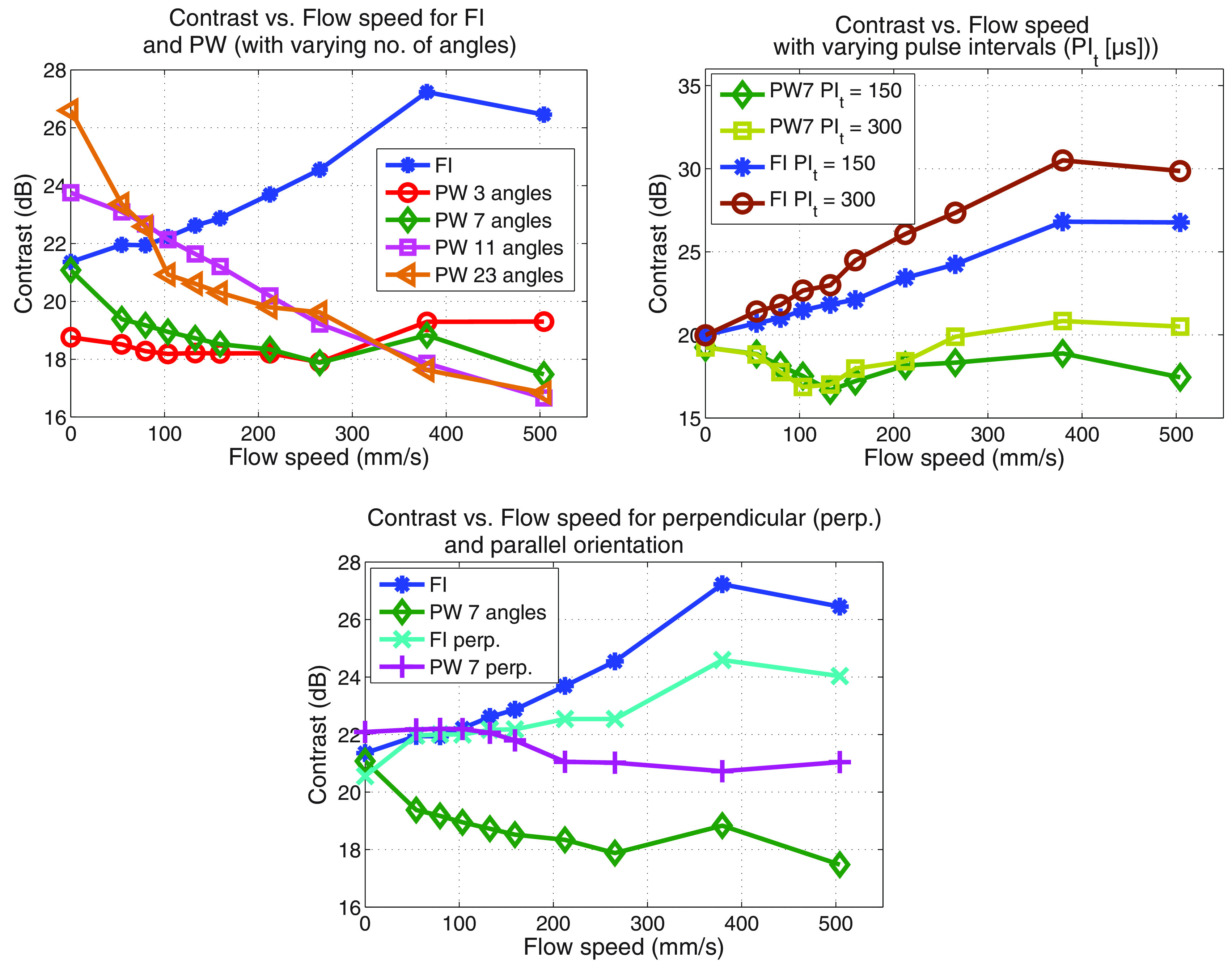
Contrast versus flow speed for (a) FI and PW imaging with varying number of angles (parallel orientation), (b) different pulse intervals (*PI*_*t*_ in $\mu$s) and (c) parallel versus perpendicular (perp.) orientation (the contrast with no flow is slightly different, due to having different ROIs for different orientations). *PI*_*t*_  =  150 $\mu$s for figure (a) and (c).

Figure 7(a) shows contrast as a function of flow speed for FI and PW with 3, 7, 11 and 23 angles. FI contrast increased and PW contrast decreased with increasing flow speed. For PW imaging, the decrease in contrast with flow speed was greater with increasing number of angles.

**Figure 8.**
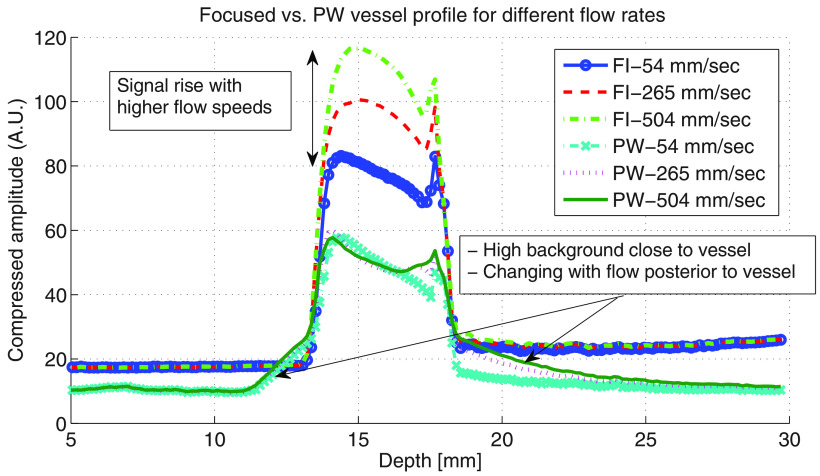
Axial amplitude profile across the diameter of the vessel for FI and PW imaging (seven angles, parallel direction). High background levels close to and posterior to the vessel are seen to increase with flow speed. Square root compression was used to illustrate the total dynamic range.

Changing the pulse interval from 0.15 ms to 0.3 ms (see figure 7(b)), the change in contrast with flow speed was increased. When the transducer was orientated perpendicular to the flow direction, the change in contrast was less for both techniques, compared to when the transducer was parallel to the flow direction (see figure 7(c)). The axial image profile through the vessel in the parallel orientation is illustrated in figure 8 which shows higher background levels in the PW image close to the vessel. The background level immediately posterior to the vessel increases with flow speed. The signal is also seen to rise inside the vessel for FI.

## Discussion

### Parameter settings

This study found that varying the MI or transmit frequency produced differences between FI and PW contrast. At the highest MI investigated, PW contrast was less than FI contrast, which is likely to be a result of signal loss due to increased bubble disruption and corresponding signal decorrelation between angles. At lower MIs, PW had a slightly better contrast than FI, probably due to its better noise cancelation. At lower frequencies, where a greater non-linear signal is expected to be generated by microbubbles closer to the resonance frequency (∼3 MHz), grating lobe artefacts, which are discussed below, reduced PW contrast.

Using a three-pulse contrast pulse sequence (CPS), Couture *et al* (2012) compared PW and FI contrast at 7.5 MHz transmit frequency and MIs in the range 0.02–0.1. Similar to the current study, they showed that PW has greater contrast than FI at lower MI, and vice versa at higher MI. However, they reported 11 dB greater contrast for PW compared to FI at 50% CR, whereas the current study was able to show that similar FI and PW contrast can be achieved at 50% CR by choosing the optimal set of parameters, as one set of values may be suitable for one mode and not for the other. Side lobe and grating lobe artefacts may contribute to loss of contrast and edge sharpness for PW imaging (figure 4 and 6). Artefacts, which could be easily observed in the perpendicular transducer orientation (figure 5(b)), were most likely high frequency grating lobes derived from the harmonics generated by microbubbles. The effect was greater at lower frequencies, which were closer to the bubble resonance frequency where more harmonics are generated and resulted in 7 dB contrast reduction (figure 6(a)) for PW imaging. The lateral profile of the artefacts (figure 6(b)) shows that they have greater intensity and are situated closer to the vessel at lower frequencies. At 3 MHz, an overall increase in noise in the axial profile (figure 6(c)) for PW is also probably due to the higher order grating lobes. Artefacts were obscured by the vessel signal in the parallel direction making them less evident, however, they resulted in loss of contrast (blurring) at the anterior and posterior vessel edges and probably false contrast signal within the vessel (figure 4). Grating lobe artefacts were not observed for FI. For the internal regions of a tumour, it is likely that vessels exist in all directions, producing artefacts that are a combination of (1) loss of contrast due to high frequency grating lobes and (2) false contrast increase and vessel edge smoothness corresponding to vessels that are parallel to the imaging plane. This may lead to degraded image quality in terms of loss of both contrast and resolution, and misinterpretation of the perfusion data. PW imaging was expected to be prone to grating lobe artefacts due to the use of the full transducer aperture for transmissions and the tilting angles. It may be worth investigating whether PW imaging with a limited aperture can be used to reduce the effect by placing the grating lobes outside the field of insonation, or whether transmit frequencies can be used where the effect is less pronounced.

### Flow effects

Contrast and vessel edge sharpness varied with speed of flow for both imaging modes. The change in apparent contrast with flow rate is believed to be due to two phenomena. First, a motion artefact, where displacement of microbubbles between pulse inversion transmissions results in false contrast signal. This happens for both FI and PW. Here, we refer to this as PI flash artefact to distinguish it from the flash artefact that appears due to tissue motion in Doppler imaging.

Second, for PW only, motion occurring between plane wave transmissions at various angles will result in lack of signal coherency during coherent summation and thus signal cancellation rather than focusing (coherent-compounding) gain. This increases with the number of angles (figure 6(a)) and was also observed in the perpendicular direction by Viti *et al* (2016). This has been previously studied by Denarie *et al* (2013) who examined the influence of rapidly moving targets on B-mode PW imaging using both simulations and *in vivo* experiments. They illustrated that axial displacements larger than half a wavelength occurring between transmissions gave lower signal to noise ratio (SNR), as well as a loss in the focusing capability of the method, i.e. lateral shifting, grating lobe formation, and hence an overall loss in resolution and contrast.

An increase in the PI flash artefact for focused imaging is observed within the vessel as flow speed increases (figure 8). However, the signal stays almost constant for PW as it is affected by the combination of the two effects. Consistent with this explanation, it was found that increasing the pulse interval led to increased artefact (figure 7(b)). Please note that the intervals between lines/angles were kept constant at twice the pulse interval. Both the PI flash artefact and incoherency would be greater, when more time is allowed for the microbubbles to move between the pulses and between transmissions for different angles.

Transducer orientation was observed to influence the effect of flow rate on contrast for both imaging modes. The difference between the lateral and elevational beam widths may be a key factor in explaining this phenomenon. Echo amplitude (and to some extent phase) at a given location in the image fluctuates as scatterers flow across the resolution volume associated with that location, and for a given flow speed the fluctuation is more rapid the narrower the resolution volume in the direction of flow (Eckersley and Bamber 2004). Since the lateral and elevational beam widths define the relevant dimensions of the resolution volume when the transducer is oriented in the parallel and perpendicular directions, respectively, for a given flow speed, the fluctuation will be more rapid in the parallel than in the perpendicular direction. Such fluctuation will cause both PI flash artefact and incoherency between different PW angles, which can therefore be expected to be more severe in the parallel than in the perpendicular direction, and this is consistent with our observations. A limitation of the current study is that flow with a component in the axial direction was not studied. It would be expected that this would cause by far the greatest effect on contrast, in part because the resolution volume is smallest in the axial direction but mostly because this is direction of wave propagation and is, by design, the direction in which the echo phase varies strongly with scatterer location. Previous studies of the effect of bubble motion on PW contrast have investigated bubble motion in the axial direction (Stanziola *et al* 2018) or elevational direction (Viti *et al* 2016). Consistent with the above hypothesis, Stanziola *et al* predicted greater reduction in contrast in the axial direction using simulations (28.3 dB) than we measured in the lateral direction (7.2 dB) using pulse inversion and 11 compounding angles. Wang *et al* (2007) simulated the effect of the motion of point targets, in both axial and lateral directions, on B-mode PW imaging signal and side lobes. Their simulations predicted that signal decreases and side lobes increase as speed increases or as the number of PW angles increases, i.e. the time to acquire one frame is greater. They also demonstrated that this effect was reduced for motion in the lateral direction. The same speeds or acquisition periods produced much less image degradation when motion was in the lateral direction compared to in the axial direction.

For PW imaging, the grating and side lobe artefacts posterior to the vessel were observed to increase with flow rate in the parallel transducer orientation (figure 8). This suggests that for echoes arising from within the grating lobes, there is false signal increase due to PI flash artefact, or a reduction in false signal loss due to PW angle incoherence, or both. When the transducer was positioned parallel to the flow direction, it is reasonable that the former would occur because for a grating lobe, there will be a substantial component of motion along the axis of sound propagation. This was not the case for the perpendicular direction and correspondingly grating lobe artefacts were not observed to change with flow rate when the transducer was orientated that way. Whatever its origin, the impact of this effect will reduce the vessel-background contrast potentially making it harder to accurately define an ROI boundary (e.g. tumour) and reduces the visibility of small vessels situated below larger vessels, possibly making them undetectable.

As discussed above, blood may flow in all directions and the effect of transducer orientation, although present locally, may average out across the imaged field. However, if there is a dominant flow direction, transducer orientation should be maintained across sequential imaging sessions. Also, maintaining the same ROI and imaging plane may be crucial in this case. 3D imaging may be used to rectify some of these considerations, such as imaging plane, however, variations in transducer orientation and ROI boundaries should be minimised. In addition, false reduction and increase in contrast for PW and FI remain an issue in 3D imaging. Although neither false reduction nor false increase in contrast is desirable, it may be more straightforward to account for such artefacts in FI as it only suffers from the PI flash effect which may in principle be compensated using flow speed. Flow correction, using fluctuation rate or/and Doppler velocimetry, is the subject of future work.

It is not easy to extrapolate our findings to centre frequencies and contrast agents other than those employed here. It can be expected that there are differences. As mentioned above, the results of our study differed from that of Couture *et al* (2012) who employed centre frequency 7.5 MHz, experimental bubbles and lower MIs. Unfortunately, it is hard to separate the effects of individual parameter settings and contrast agent to help us understand why their results differed from ours. We do expect that some of the same trends we observed in the current study will be present for other transducers. Specifically, higher peak negative pressure and greater F# will give higher bubble disruption and therefore PI flash artefact irrespective of transducer. More disruption might be expected at frequencies lower than those used here, as one approaches the resonant frequency of most of the microbubbles in a typical population (Forsberg *et al* 2005). It can also be expected that other transducers will suffer motion artefacts due to loss of coherency between compounding angles. Indeed, Stanziola *et al* (2018) also observed these effects using a phased array. Irrespective of transducer, choosing a more stable contrast agent is clearly desirable to minimise both PI flash artefact and reduction of coherency between angles.

The overall aim of our work is to develop a CEUS imaging system. An advantage of PW imaging is faster acquisition speeds, however, if fast imaging is not required, based on our observations, focused imaging is a better choice providing greater image quality for similar rates of contrast reduction. One example where fast imaging rates may be advantageous is 3D DCE-US. Fast 3D imaging would allow adequate sampling of the time intensity curve if averaged over a volume. However, if a mechanically swept transducer is used, the volume rate is likely to be limited by the sweep speed of the transducer and focused imaging frame rates will provide adequate spatial sampling of the volume of interest. Plane wave imaging can be reconsidered in future if a 2D matrix array is used to achieve greater volume rates.

## Conclusions

The effect of imaging modes and imaging parameters, flow speed and flow direction were evaluated on vessel contrast for focused imaging and plane wave imaging *in vitro*. Overall, this study showed that, with careful choice of parameters, similar contrast is achievable at a similar contrast reduction rate for focused and plane wave imaging. The study also showed that plane wave imaging suffered from high frequency grating lobe artefacts which may lead to false increase or decrease of the contrast and smoothed vessel edges.

Both focused and plane wave imaging contrasts were influenced by flow rate which caused motion artefact, referred to as PI flash artefact. This effect, and that of PW angle incoherency, may be negligible for capillaries where the flow is very slow (up to 1 mm s^−1^) but for veins or arteries, the effects may create false contrast for focused and plane wave imaging or reduced contrast for plane wave imaging.

Based on our observations, if fast imaging is not required, focused imaging is a better choice providing greater image quality for similar rates of contrast reduction. Plane wave imaging can be reconsidered in future if methods are developed for plane wave artefact compensation.
